# Experience, Perceived Knowledge and Perspectives of Physiotherapists Towards the Use of Virtual Reality-Based Interventions in the Musculoskeletal Setting: A Cross-Sectional Survey

**DOI:** 10.7759/cureus.85093

**Published:** 2025-05-30

**Authors:** Fady Salama, Joachim Ho, Rokhsaneh Tehrany, Peter Snow

**Affiliations:** 1 Division of Medical Sciences, University College London, London, GBR; 2 Department of Medicine, University Hospital Coventry and Warwickshire, Coventry, GBR; 3 Department of Physiotherapy, Royal National Orthopaedic Hospital, London, GBR; 4 Department of Orthopaedics and Musculoskeletal Science, Royal National Orthopaedic Hospital, London, GBR

**Keywords:** musculoskeletal conditions, neuro-visual rehabilitation, pain, physiotherapy, virtual reality

## Abstract

Background

Multimodal physiotherapy including exercise therapies is widely advocated for people with musculoskeletal (MSK) conditions; however, many people continue to experience persistent pain. Virtual reality (VR) based interventions involving exercise provision within a highly engaging virtual environment may provide opportunities for MSK rehabilitation. Since physiotherapists will play a key role in implementing VR interventions in the future, this pilot study aimed to explore UK physiotherapists’ knowledge, experience and perceptions of VR-based interventions for MSK rehabilitation, with a particular focus on identifying barriers and facilitators to implementation in addition to informing the design/conduct of a larger survey in the future.

Methods

A cross-sectional online pilot survey was conducted using convenience sampling between June and August 2023. Eligible participants included UK physiotherapists working across any clinical, research, or educational setting. Data were analysed descriptively using means, percentages and frequency distributions.

Results

From the valid 40 responses, most were practising physiotherapists (n = 33, 83%) specialising in the MSK field (n = 34, 85%). The majority had little familiarity with VR (n = 21, 53%) and knew little about VR for pain management (n = 23, 58%). A significant proportion had engaged with VR for entertainment (n = 17, 43%), while fewer had done so for research purposes (n = 7, 18%). If available, nearly half agreed that they would like to offer VR for MSK rehabilitation (n = 19, 48%) and also agreed that patients might be willing to engage with VR interventions (n = 22, 55%). ‘Cost of purchase and maintenance’ and ‘clinician familiarity’ were ranked as the most important barriers to implementation.

Conclusion

Although physiotherapists have limited knowledge/experience of VR-based interventions for MSK pain management and identify barriers to implementation, positive perceptions towards the intervention were expressed. A more extensive survey, in addition to qualitative investigations, is needed to generalise these preliminary findings to the wider physiotherapy workforce.

## Introduction

Musculoskeletal (MSK) conditions involving the joints, muscles and bones affect around a third (20.3 million) of the UK population and are a leading cause of chronic pain and physical disability around the world. Amongst all MSK conditions, neck, lower back and osteoarthritis have the highest UK prevalence, which increases with age, sedentary lifestyles and obesity [1]. While MSK conditions are pathophysiologically heterogeneous, chronic MSK pain, which is defined as pain lasting more than three months, is a leading symptom common to all conditions. In the UK, around 28 million live with chronic MSK pain [2]. These people often have a diminished sense of psychological well-being and quality of life due to factors such as reduced mobility and functional impairments, which limit the ability to perform routine activities of daily living or engage in social or occupational roles. In light of the surge in factors contributing to chronic MSK pain, including the aging population and rise in sedentary lifestyles following a shift to home-based work patterns, the UK is facing a major public health crisis, with long waiting times for community MSK assessment and the greater use of healthcare resources [3]. These observations necessitate the need to optimise strategies for managing MSK conditions. 

Although the management of many MSK conditions involves a multidisciplinary approach consisting of a combination of pharmacological, non-pharmacological and surgical interventions, common to the management of all MSK conditions is referral for multimodal physiotherapy [4]. Physiotherapy broadly aims to restore physical activity and movement and enhance strength and endurance through a combination of self-management strategies and guided exercises, many of which have been recommended within national guidelines due to the clinical and cost-effectiveness [5]. While there are varying levels of evidence for each component, there is a growing consensus on the efficacy of exercise therapy, which is now widely considered a cornerstone of MSK rehabilitation [6]. However, many people with MSK conditions continue to experience persistent pain in the absence of any meaningful improvement [7]. A significant determining factor to the success of exercise therapies relates to adherence to prescribed exercise programmes, which includes appointment attendance and correct performance of exercise [8]. Poor engagement with exercise therapy has adverse effects on outcomes, correlates with increased healthcare costs and is associated with various physical, psychological and clinical barriers, including low self-efficacy and heightened levels of baseline depression [9]. Interventions which enhance engagement with prescribed physiotherapy are therefore of great interest.

Virtual reality (VR) technologies create simulated virtual environments, which can be viewed through immersive headsets (goggles) or non-immersive computer screens [10]. Within these manipulated virtual environments, it is possible to interact with objects, games and tasks as a ‘virtual character’ or ‘avatar’, creating multiple opportunities for rehabilitation and pain management due to the ability to personalise these environments to suit individual needs. Although the ways in which VR facilitates pain relief is not fully understood, multiple theories have been proposed, including altering the neural pain control system [11], distraction [11], enhanced engagement [12] and creating illusions beyond distractive analgesia through immersion and high interactivity [12]. To date, VR has demonstrated a supporting role in many conditions and settings, such as burns, strokes, Parkinson’s disease and more [13]. More recently, the use of VR-supported rehabilitation for the management of orthopaedic/MSK conditions has been the focus of systematic reviews [14-16]. While common to all these reviews has been the absence of high-quality randomised controlled trials (RCTs), VR has demonstrated potential within the MSK field.  Although the evidence base demonstrating the clinical and cost-effectiveness of VR is very much in its infancy within the MSK setting, we might expect to see a rise in studies evaluating outcomes and use within clinical practice in light of the increasing adoption and accessibility of off-the-shelf commercial VR headsets and software [17].

To support the broader adoption of VR-based interventions within the MSK setting in the future, it is crucial to gain buy-in from clinical stakeholders, particularly physiotherapists, who play a key role within rehabilitation services. Physiotherapists and their support staff possess key insights into the practical feasibility of new interventions, particularly with regards to how they integrate into existing clinical practice and routines. Many technology-based healthcare interventions fail to reach clinical adoption because the primary focus is the technology itself, without adequate consideration of how it will fit into existing clinical routines; hence, understanding their perspectives, experiences and knowledge may help identify early practical barriers to implementation to inform the future development [18]. Recent studies have explored physiotherapists’ perspectives on the use of VR in MSK rehabilitation. Brady et al., through qualitative focus groups, highlighted physiotherapists’ cautious optimism towards VR for shoulder pain rehabilitation, particularly its potential to manage kinesiophobia and enhance patient engagement, while also raising concerns around safety, patient selection and integration into clinical workflows [19]. Similarly, Felsberg et al. conducted a large-scale survey of U.S. physical therapists and physical therapist assistants, revealing that while current adoption of VR remains low, positive attitudes, perceived ease of use and workplace support were associated with greater intention to adopt VR in future practice [20]. However, despite the emerging interest surrounding the perspectives of physiotherapists towards VR-based intervention, research on the topic remains in its early stages. Gaining a broader insight into UK-specific perspectives, experiences and perceived knowledge will help researchers design VR interventions that are well-suited to the UK healthcare system, with both patients and staff in mind.

The present study builds on previous findings on the topic through a UK-wide, survey- focused on chronic MSK pain across a range of clinical contexts. The primary aim of this pilot study was to assess the feasibility for the survey design and to evaluate the perceived knowledge, experience and perceptions of the physiotherapy workforce towards VR-based interventions in the MSK setting, with a particular focus on identifying barriers to implementation to inform future research.

## Materials and methods

Design

A cross-sectional online pilot survey was conducted to explore physiotherapists’ perceived level of knowledge, experience and perceptions towards the use of VR-based interventions for MSK pain management and rehabilitation. Ethical approval was granted by the University College London (UCL) Research Ethics Committee in June 2023 (ref: 6676/004), and the study was reported according to the Strengthening the Reporting of Observational and Epidemiological studies (STROBE) statement [18].

Questionnaire development

The questionnaire was developed through and expert consultation and piloting phase. A literature search identified key areas of interest, which were refined through discussion amongst the authors (a physiotherapist, healthcare engineer and medical students). The initial questionnaire consisted of 43 questions and four sections including participant demographics/clinical experience, perceived level of knowledge, experience and perceptions towards VR-based interventions. Questions included open text box questions, multiple choice and ranking of statements according to five-point Likert scales.

After the initial consultation phase amongst the research team, the questionnaire was further refined through consultation with a panel of independent physiotherapists (n=4), who specialised in the field of MSK rehabilitation. Feedback was provided to shorten the questionnaire, improve readability and enhance the conciseness of the question, as well as to reduce questionnaire fatigue. The final questionnaire consisted of 33 items, which were developed into an online Microsoft Teams form (Appendix A). The online form was then piloted by four UCL students and three physiotherapists to ensure the collection of meaningful data, as well as to measure average completion time to inform recruitment strategies.

Participants

Potential participants were recruited over a three-month period, between June and August 2023. The inclusion criteria were individuals who were studying or working within the physiotherapy workforce, including those across clinical practice, research, education, or leadership and management roles. To participate, individuals were required to be based in the UK at the time of the study, aged 18 or over and able to provide informed consent for their participation and for the storage and use of their data in accordance with UCL ethics policies.

Recruitment

Convenience sampling was used to recruit physiotherapists, students or physiotherapy technicians working across the clinical, education, research or leadership field as the study was interested in exploring all views amongst the physiotherapy workforce. The online survey platform Microsoft Forms was used to host the questionnaire before several recruitment strategies were implemented to disseminate the survey link.

Recruitment strategies involved dissemination of the survey link and recruitment posters via social media platforms (‘X’ (formerly known as Twitter) and ‘LinkedIn’). Within the X platform, a study handle (@VRsurveystudy) was registered to post the initial recruitment materials/posts. Relevant hashtags relating to ‘VR’ and ‘physiotherapy’ were embedded within the posts to maximise their reach. Multiple accounts that were linked to the physiotherapy profession, such as the Chartered Society of Physiotherapy CSP) and the authors’ X handles (@rmtehrany and @peterwsnow, were also tagged to encourage wider dissemination/promotion of the posts to their following networks through snowballing. The survey was also directly disseminated through the CSP by posting the study synopsis and survey link within the news section of the ‘Musculoskeletal’ and ‘Orthopaedics’ networks.

Upon clicking on the survey link, potential participants were provided with an opportunity to read the Participant Information Sheet (Appendix B) and discuss their participation with relevant others. If they wished to participate, they were then guided to a series of screening questions to check eligibility and provide consent. Once consent was given, participants were then directed to the main survey questions. Participants were free to withdraw answers up until the time of submission. After this time point, all submitted answers could not be retracted, which was clearly stipulated during the consent process.

Variables and statistical analysis

While a minimum of 100 responses has been recommended for surveys, with 33 participants needed per subgroup, this was a pilot survey in order to inform a larger study and was therefore not hypothesis-driven. For pilot studies, a sample size of 12-40 responses has been recommended [21]. Questionnaire responses were collected through Microsoft Forms and subsequently exported into Microsoft Excel for analysis. To minimise missing data, all multiple-choice questions were mandatory. Responses from multiple-choice questions and Likert scales were summarised using descriptive statistics (frequency distributions and percentages). Data from open text responses were also counted and summarised descriptively using frequency distributions. Subgroup analysis using inferential statistics was not deemed appropriate as this was a pilot study aiming to provide preliminary data and test the feasibility of the methods rather than to test specific hypotheses. Furthermore, as the sample size was not large enough, the results would be at risk of type 2 errors.

## Results

Participant characteristics 

A total of 46 participants initially responded to the survey. After screening, 40 responses were valid and subsequently included in the analysis. It was not possible to determine the response rate since the survey was snowballed through social media platforms and professional organisations [19].

Most respondents identified as female (72.5%), were aged between 41-50 years of age (37.5%) and worked within England (95%). In terms of job description, the majority were ‘practising clinical physiotherapists’ (82.5%) who specialised within the MSK field (85%) and had over 20 years of post-qualification work experience (37.5%). Amongst the respondents who worked for the NHS (85%), most were based within the tertiary healthcare sector (50%) and were ‘advance practitioners’ working at a band 8a level (32.4%). In terms of educational qualifications, most were educated to a BSc (or equivalent) (45%) followed by MSc level (42.5%) (Table 1).

**Table 1:** Participant characteristics, clinical experience and place of work *Questions where multiple responses were possible. **Questions exclusive to respondents who indicated that they worked within an NHS setting. NHS Bands refer to the pay grading structure within the NHS in the UK and also depicts the level of authority, proficiency and duties assigned to a person. Band 4 represents entry-level positions, Band 5 is for newly qualified physiotherapists, Band 6 for experienced roles, Band 7 for advanced clinical or managerial roles and Band 8 for senior leadership positions. ***Questions exclusive to respondents who indicated they worked in England.

Category	Sub-category	Responses (n)	Percentage (%)
Age range (years) (n=40)			
21–30		13	32.5
31–40		7	17.5
41–50		15	37.5
51–60		3	7.5
61–70		2	5
Total responses		40	-
Gender (n=40)			
Male		11	27.5
Female		29	72.5
Total responses		40	-
Years of experience since qualifying (n=40)			
2–5 years		8	20
5–10 years		8	20
10–20 years		8	20
>20 years		15	37.5
Not applicable		1	2.5
Total responses		40	-
Highest professional qualification (n=40)			
BSc or equivalent		18	45
PGDip		3	7.5
MSc		17	42.5
PhD		2	5
Total responses		40	
Job description (n=40)*			
Practicing clinical physiotherapist		33	82.5
Physiotherapist assistant/technician		1	2.5
Clinical academic		6	15
Physiotherapy researcher		6	15
Physiotherapy educator		4	10
Non-clinical physiotherapy leader		1	2.5
Total responses		51	-
Primary area of expertise (n=40)			
Neurology		2	5
Musculoskeletal		34	85
Orthopaedics		1	2.5
Chronic/persistent pain		2	5
Other		1	2.5
Total responses		40	-
Primary work setting (n=40)			
NHS		34	85
Private sector (company)		2	5
Higher education institute/university		2	5
Other		2	5
Total responses		40	-
Type of NHS setting (n=34)**			
Community care		2	5.9
Primary care		8	23.5
Secondary care		7	20.6
Tertiary care		17	50
Total responses		34	-
Professional NHS band (n=34)**			
Band 4		1	2.9
Band 6		8	23.5
Band 7		8	23.5
Band 8a		11	32.4
Band 8b		3	8.8
Band 8c		2	5.9
Band 8d		1	2.9
Total responses		34	-
Country of primary work (n=40)			
England		38	95
Scotland		1	2.5
Wales		1	2.5
Total responses		40	-
Region of primary work (n=38)***			
London		26	68.4
Northwest		2	5.3
Southeast		3	7.9
Southwest		2	5.3
West Midlands		3	7.9
Yorkshire and the Humber		2	5.3
Total responses		38	-

Perceived knowledge 

Table 2 presents the percentage of ratings in response to questions that asked about respondents' level of perceived knowledge of VR and VR-based interventions, both generally and within the context of pain management and rehabilitation. Most respondents reported that they had ‘little’ familiarity with the term ‘virtual reality’ (52.5%) and even less (very little) familiarity with the difference between immersive and non-immersive VR interventions (40%). The majority of respondents (57.5%) also knew ‘little’ about the use of VR as a therapeutic intervention for pain management and rehabilitation, as well as ‘little’ (40%) about the ways/mechanisms VR works on to facilitate pain relief.

**Table 2:** Participant ratings on perceived knowledge of VR and VR-based interventions for pain management and rehabilitation Mode refers to the most frequent response obtained. VR, virtual reality

Question	Not at all, n (%)	Very little, n (%)	Little, n (%)	Somewhat, n (%)	Extremely, n (%)	Mode
How familiar are you with the term ‘virtual reality’? (n=40)	0 (0)	3 (7.5)	21 (52.5)	12 (30)	4 (10)	Little
How familiar are you with the difference between ‘immersive and non-immersive’ virtual reality? (n=40)	6 (15)	16 (40)	9 (22.5)	6 (15)	3 (7.5)	Very Little
How familiar are you with the use of ‘virtual reality’ as a therapeutic intervention for pain management and rehabilitation? (n=40)	6 (15)	8 (20)	23 (57.5)	2 (5)	1 (2.5)	Little
Do you know of any ways/mechanisms virtual reality works on to reduce pain? (n=40)	10 (25)	12 (30)	16 (40)	2 (5)	0 (0)	Little

**Figure 1:**
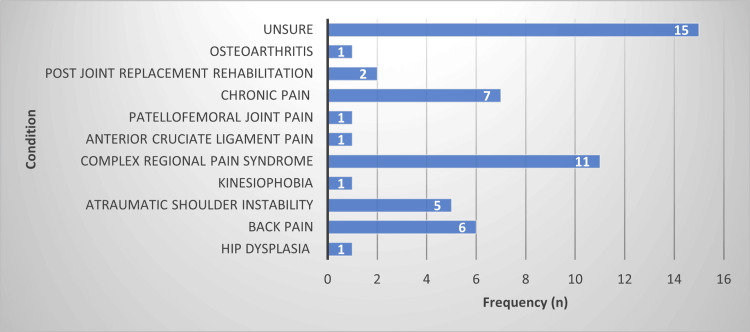
Summary of open text box answers in response to question 20: ‘name/describe any MSK conditions that have previously been managed using VR-based interventions’ (total respondents, n = 40). MSK, musculoskeletal; VR, virtual reality

Respondents were asked to provide open text box answers in response to a question asking them to ‘name/describe any MSK conditions that have previously been managed using VR-based interventions’. The responses from the open text box answers have been collated and presented in Figure 1. In total, 40 respondents answered this question. While 15 respondents reported that they were ‘unsure’, the most commonly cited condition was complex regional pain syndrome (CRPS) (n=11), followed by chronic pain (n=7).

Previous experience and exposure to VR and VR-based interventions 

Based on the findings in Table 3, when asked about previous experience of engaging with VR, most respondents reported that they had no exposure to any form of VR (40%) or had only engaged for personal entertainment purposes (42.5%). Of the fewer respondents who had previous experience in delivering VR as part of a treatment intervention or had engaged with VR as part of a research project (n=9), the majority went on to indicate that their role was part of a research team conducting VR-related research (55.6%). The hospital inpatient (33.3%) or university setting (33.3%) were cited as the most common VR settings for research and clinical delivery purposes. Furthermore, the majority of this sub-group predominantly reported scientific conferences (44.4%) as a source of training/knowledge acquisition.

**Table 3:** Frequency of responses to questions about previous experience and exposure to VR-based interventions ^a^Question open to respondents who indicated they had been involved with ‘delivering VR as a treatment intervention’ or ‘engaged with VR as part of a research project’. ^b^Questions where multiple responses were possible. VR, virtual reality

Questions and sub-categories	n	%
Do you have any experience of engaging with VR? (n=40)		
I have not had any experience of engaging with any form of VR	16	40
I have engaged with VR for personal entertainment purposes	17	42.5
I have been involved with delivering VR as a treatment intervention for patients	5	12.5
I have engaged with VR as part of a research project	7	17.5
Other: ‘education’	1	15
Total number of responses	46	-
What was your role?^a,b^ (n=9)		
A research participant	1	11.1
Part of a research team	5	55.6
Responsible for delivering a VR intervention in a study	1	11.1
Supporting a clinician to deliver a VR intervention in a study	1	11.1
The clinician delivering a VR intervention in a clinical setting	2	22.2
Supporting a clinician to deliver a VR intervention in clinical practice	2	22.2
Other: ‘student project on placement’	1	11.1
Other: ‘referred to VR rehab intervention’	1	11.1
Total number of responses	14	-
What setting was the VR intervention used in?^a^ (n=9)		
Hospital outpatients setting	2	22.2
Hospital inpatient setting	3	33.3
University/academic institute	3	33.3
Other: ‘multiple’	1	11.1
Total number of responses	9	-
Have you ever engaged with any training opportunities relating to VR as an intervention for pain management and rehabilitation?^a,b^ (n=9)		
Yes, at undergraduate level	1	11.1
Yes, at postgraduate level	1	11.1
Yes, as part of a workshop	2	22.2
Yes, as part of my work in-service training	1	11.1
Yes, as part of a conference	4	44.4
No	3	33.3
Total number of responses	12	-

Perceptions towards VR-based interventions for MSK pain management and rehabilitation 

Based on the answers presented in Table 4, if available, the majority (47.5%) of respondents ‘agreed’ that they would like to offer VR as an intervention for MSK rehabilitation and pain management. Most also ‘agreed’ (55%) or ‘strongly agreed’ (22%) that patients would be ‘willing to engage with VR-based interventions if available to them’. Similarly, positive attitudes were also expressed in response to statements asking about perceptions towards training opportunities. The majority of respondents either ‘agreed’ (57.5%) or ‘strongly agreed’ (30%) that they would like to see more training opportunities on the use of VR-based interventions for MSK pain management and rehabilitation.

**Table 4:** Participant ratings of perceptions towards of VR and VR-based interventions for pain management and rehabilitation Mode refers to the most frequent response obtained. VR, virtual reality

Question	Strongly disagree, n (%)	Disagree, n (%)	Undecided, n (%)	Agree, n (%)	Strongly agree, n (%)	Mode
If available, you would like to offer virtual reality as an option for MSK pain management and rehabilitation? (n=40)	0 (0)	2 (5)	10 (25)	19 (47.5)	9 (22.5)	Agree
You would like to see more training opportunities on the use of virtual reality interventions for musculoskeletal pain management and rehabilitation? (n=40)	0 (0)	0 (0)	5 (12.5)	23 (57.5)	12 (30)	Agree
You anticipate patients would be willing to engage with virtual reality if the intervention was routinely available as a treatment option for musculoskeletal pain management and rehabilitation? (n=40)	0 (0)	3 (7.5)	6 (15)	22 (55)	9 (22.5)	Agree

Perceived barriers towards the implementation of VR interventions within the MSK setting  

**Figure 2:**
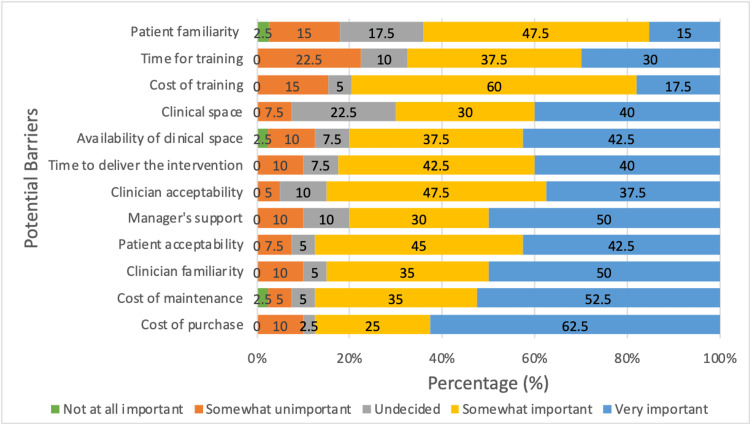
Barriers to implementing VR-based interventions in the MSK setting: percentage of ratings (total respondents, n=40). MSK, musculoskeletal; VR, virtual reality

From a list of 12 potential barriers associated with implementing VR-based interventions within routine clinical practice for MSK rehabilitation and pain management, respondents were asked to rate the perceived level of importance of each barrier against a five-point Likert scale (1=not at all important, 5=very important). Figure 2 demonstrates that all potential barriers listed were mostly considered as ‘somewhat important’ or ‘very important’. The barriers perceived as ‘most important’ were ‘cost of purchase’ (62.5%) and ‘cost of maintenance (52.5%). In contrast, the barriers most frequently ranked as ‘somewhat unimportant’ were ‘time for training’ (22.5%), ‘patient familiarity’ (15%) and ‘cost of training’ (15%).

Open text box responses: views of MSK conditions amenable to VR-based interventions

**Figure 3:**
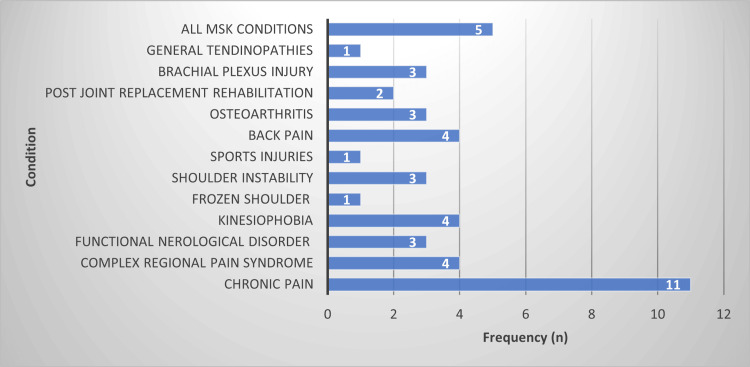
Summary of open text box answers in response to question 31 ‘name/describe any conditions you would perceive would be amenable to VR-based interventions (total respondents, n=40). VR, virtual reality

Respondents were asked to provide open text box answers in response to question 31: ‘name the musculoskeletal condition(s) that you anticipate would benefit from VR-based interventions’, where the answers were not mutually exclusive. Out of a total of 45 responses, a wide range of MSK conditions were recorded, where the most commonly cited conditions were ‘chronic pain’ (n=11), ‘all MSK conditions’ (n=5), ‘back pain’ (n=4), ‘kinesiophobia’ (n=4) and ‘CRPS’ (n=4) (Figure 3).

## Discussion

This pilot study set out to evaluate the feasibility of the survey design and explore UK physiotherapists’ knowledge, experience and perceptions towards VR-based interventions for MSK pain management and rehabilitation to inform future research. The results indicate that most physiotherapists had little familiarity with VR-based interventions in a clinical setting, especially regarding its role within rehabilitation and pain management. A large proportion of respondents had not had any experience of using VR, and of those who did, only a small proportion had experience of engaging with it as a clinical intervention. Although these findings may not be representative of the wider physiotherapy workforce since the majority of the respondents were ‘advanced practitioners’, working at a Band 8a or above with more than 20 years of clinical experience, these preliminary findings are perhaps unsurprising since VR-based interventions have not yet achieved widespread clinical adoption in the UK healthcare setting. Furthermore, in parallel to the increasing availability of affordable VR technologies, such as ‘off-the-shelf headsets’ [22], there has been a rise in interest over the last decade in its utility for the management of MSK conditions [23]. Some conditions, such as lower back pain, have been targeted more extensively using VR [11]; however, high-quality RCTs demonstrating the clinical and cost-effectiveness of VR for other common MSK conditions, such as MSK shoulder pain, are lacking [14].

Despite the limited knowledge surrounding VR, perceptions towards VR-based interventions in the MSK setting were mostly positive, mirroring findings from previous research [19]. Nearly half of respondents agreed that they would be open to offering VR-based interventions in the future, and over half believed that their patients would be too. In the context of the aging population and the rise of chronic MSK conditions [24], the demand for better MSK healthcare and rehabilitation remains as significant as ever. Physiotherapy-led exercise therapy remains the cornerstone of many condition-specific guidelines, including lower back pain, neck and shoulder pain [19]. Hence, physiotherapists might foresee the potential merits of delivering traditional exercise-based therapies within a personalised immersive virtual environment [15,24]. By wearing the headset, the user can interact with the virtual environment as an avatar (virtual character), offering several potential benefits for rehabilitation. These include enhancing motivation and adherence to structured exercise programs through the provision of real-time feedback, distraction from pain, manipulation of joint position sense and graded exposure. Kinesiophobia (fear of movement) is a common barrier to recovery amongst people with MSK conditions due to avoidance behaviours to certain movements, which are beneficial to recovery. With time, kinesiophobia leads to reduced range of movement, stiffness, and weakness; hence, it is associated with high levels of pain intensity and disability [25]. The management of kinesiophobia involves a combination of psychological and physical strategies, such as graded exposure to movement and distraction, all of which can be achieved through VR. By managing kinesiophobia, people are more likely to adhere to their exercise programs to achieve better outcomes. Other advantages include the ability to enhance motivation. By immersing people in engaging, interactive environments, VR makes exercise feel like a game or adventure, reducing boredom associated with the repetitive nature of traditional exercises. Gamification of exercises not only makes exercise therapy more enjoyable but also provides real-time feedback on performance, which can aid in goal setting and facilitate the ability to track progress. Outside the MSK setting, VR has already demonstrated effectiveness for multiple settings and conditions, including (but not limited to) wound care, chemotherapy and medical procedures [11,12].

Personal beliefs towards the intervention, motivation towards change and level of skill and confidence have previously been cited as potential challenges to achieving implementation of hospital-based interventions [26,27]. While the findings from the current study provide an indication that physiotherapists hold positive perceptions towards VR in the context of pain management and rehabilitation, many operational barriers to implementation were perceived as ‘highly important’. Amongst the 12 pre-defined potential barriers, cost of purchase and maintenance, clinician familiarity and manager support were ranked as the most important. Although the NHS is actively promoting digital transformation, such as through the expansion of virtual care and early intervention, these insights suggest that awareness regarding the potential benefits of VR in terms of clinical and cost-effectiveness may also be currently underdeveloped, mirroring the findings that respondents largely had little familiarly with VR as a healthcare intervention. These observations underscore the importance of providing education and training on the topic, particularly as digital transformation remains at the forefront of government agendas [28]. The findings are therefore of relevance to clinical and operational managers.

Furthermore, these insights highlight the need for physiotherapist to be actively involved in the development of future interventions, as they will be key to facilitate the delivery of these interventions, and they hold valuable clinical knowledge/skillset that could enhance clinical adoption. In the shorter term, however, future qualitative research is needed to explore potential barriers to implementation in greater detail.

Strengths, limitations and future research

To the author’s knowledge, this is the first survey to explore the perceived knowledge, experience and perspectives of physiotherapists towards VR-based interventions in the context of MSK pain management and rehabilitation. These preliminary findings are therefore considered novel and present opportunities for further investigation through a more extensive future survey. One of the study’s strengths lies in the development of the questionnaire, which was designed collaboratively by a multi-professional team of healthcare engineers and physiotherapists specialising in MSK rehabilitation.

The study had some limitations. Firstly, as this was a pilot study where it was not possible to conduct inferential statistics; hence, the findings are not confirmatory, warranting further investigation. Secondly, the findings should be interpreted with caution due to the small sample size, which predominantly comprised of senior clinicians at Band 8a or above, limiting the generalisability of the results. The views expressed may therefore not represent the wider physiotherapy workforce, and convenience sampling may have contributed to over representation from more experienced clinicians. While dissemination of the survey through social media and professional organisations can be effective for reaching wider communities, there is a potential to introduce sampling bias and geographic clustering [29], particularly in London. The reliance on participants having internet access and active social media profiles may also have unintentionally excluded certain groups [30]. A future, more extensive survey utilising purposeful sampling which is disseminated through NHS gatekeepers might attract a more diverse sample representative of the wider physiotherapy workforce. Additionally, specific focus should be placed on the perspectives of physiotherapist technicians, as well as physiotherapists, as they are key in the implementation of physiotherapist-designed regimes, particularly working within the NHS, as their experiences and views may differ from those in private settings.

## Conclusions

In summary, the preliminary findings suggest that physiotherapists hold positive perceptions regarding the potential of VR-based interventions in the context of MSK pain management and rehabilitation; however, their experience and perceived knowledge are limited in comparison. As the physiotherapy workforce will likely play a key role in the delivery of these interventions in the future, targeted education to increase awareness about the benefits and capabilities of VR is warranted in the short term, in addition to developing VR interventions in partnership with physiotherapists and patients in order to overcome some identified barriers to implementation. A future more extensive, NHS-wide survey is needed to confirm these preliminary findings in addition to qualitative interviews to obtain a deeper understanding of the topic.

## Figures and Tables

**Figure 4:**
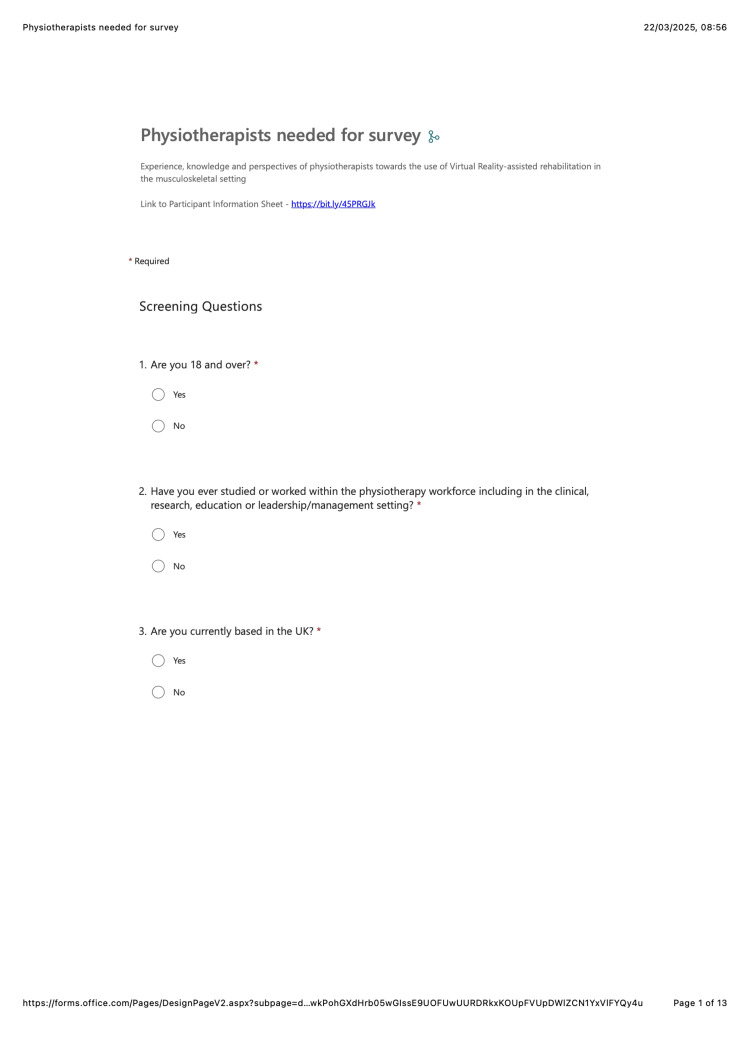
Survey page 1

**Figure 5:**
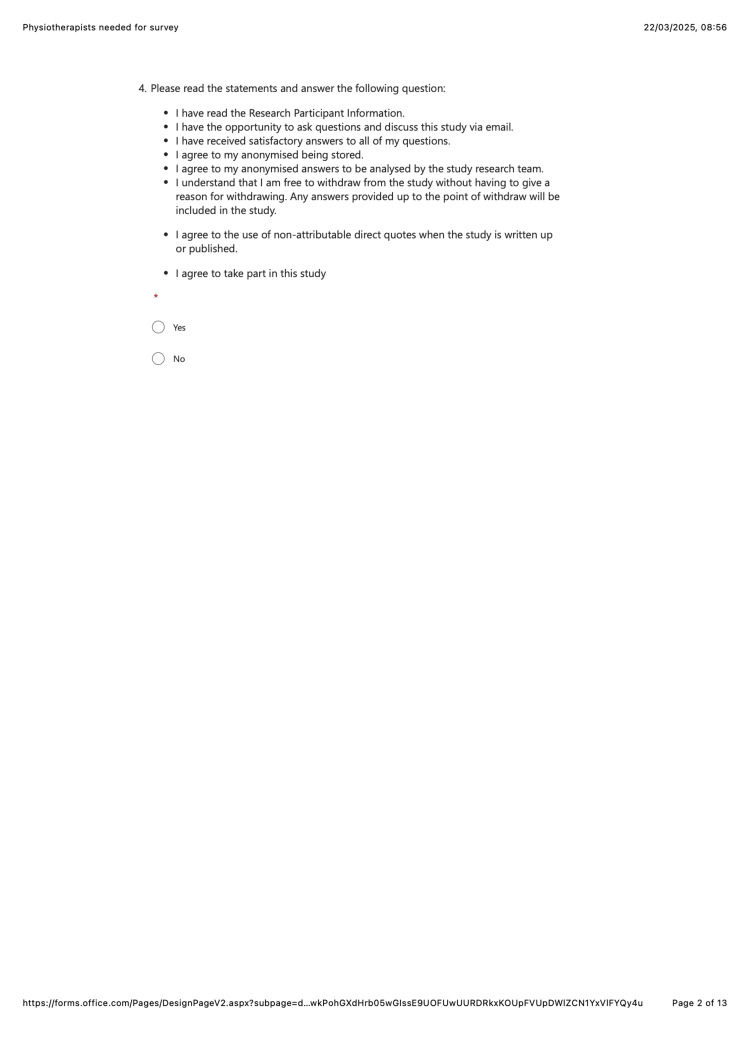
Survey page 2

**Figure 6:**
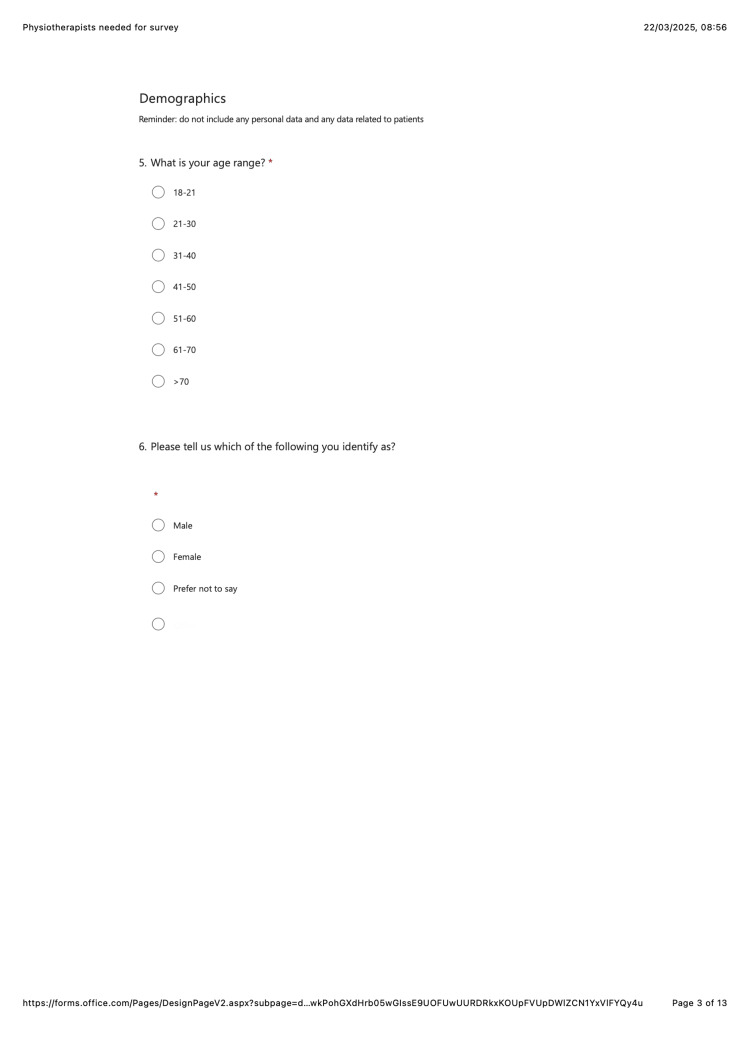
Survey page 3

**Figure 7:**
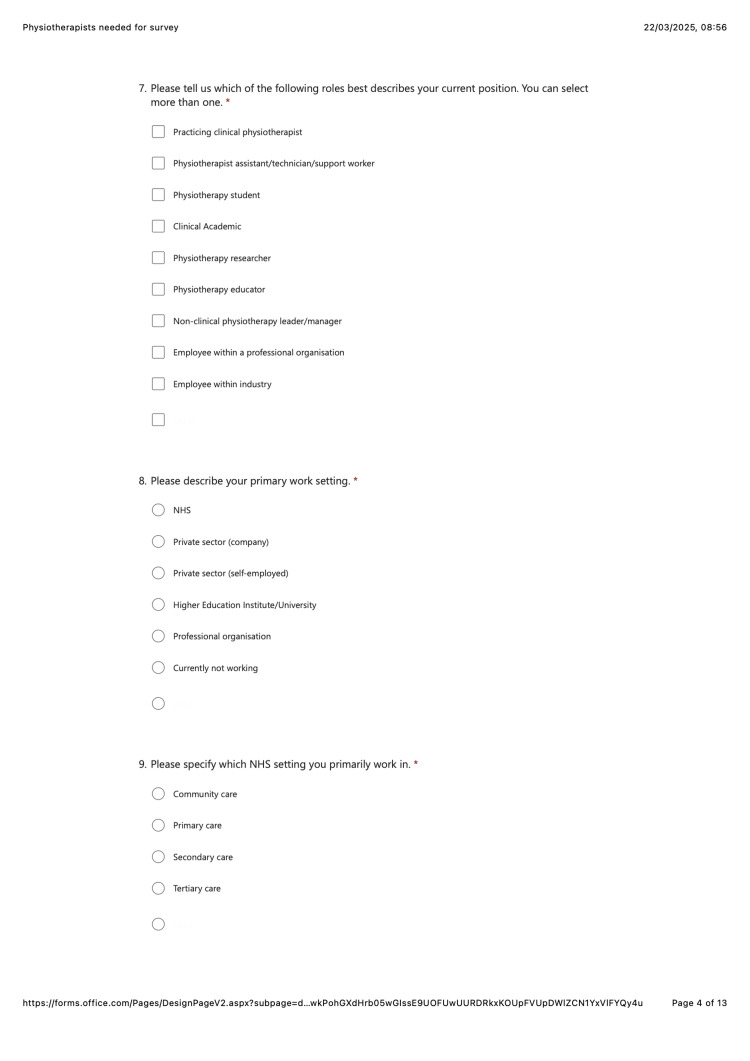
Survey page 4

**Figure 8:**
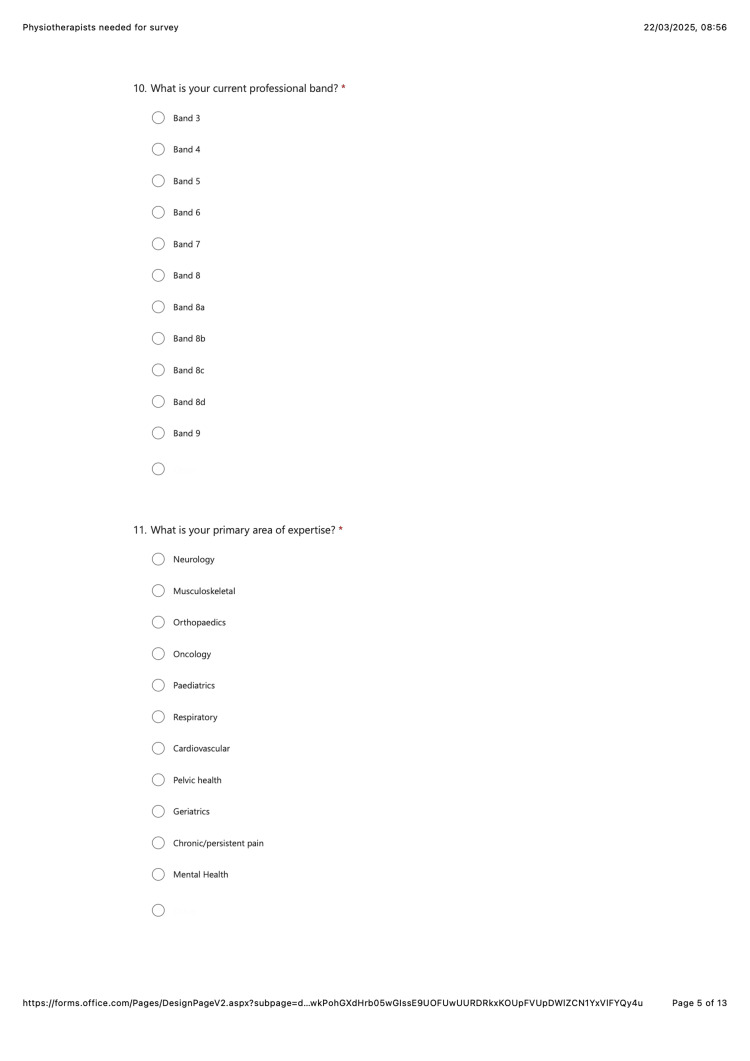
Survey page 5

**Figure 9:**
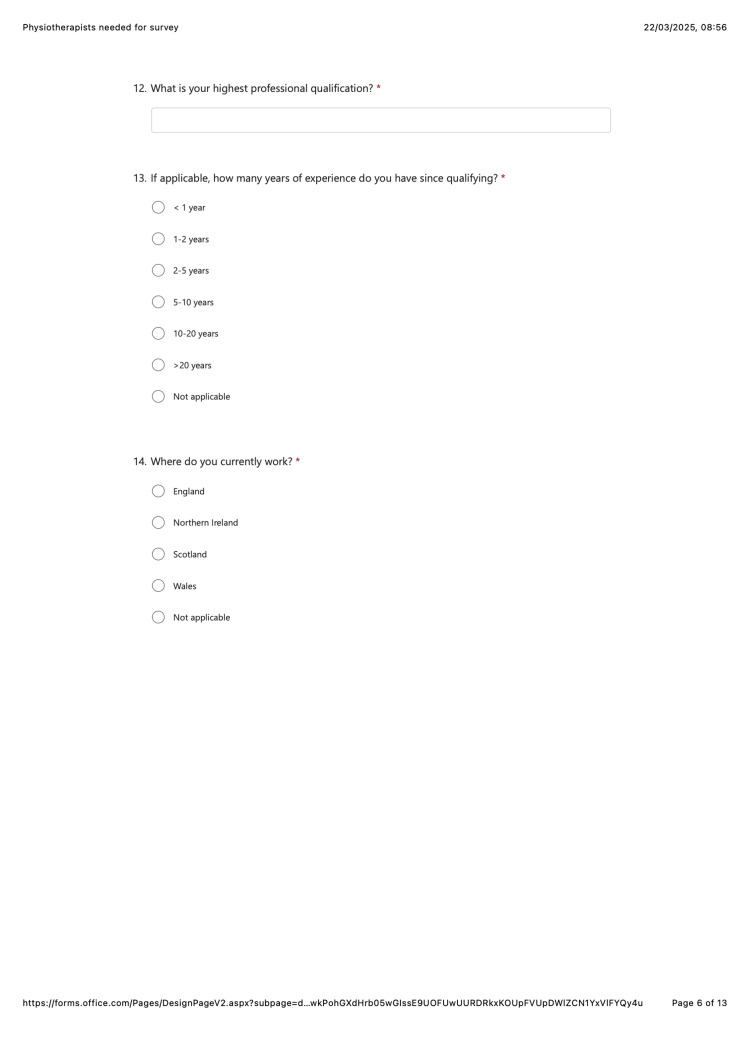
Survey page 6

**Figure 10:**
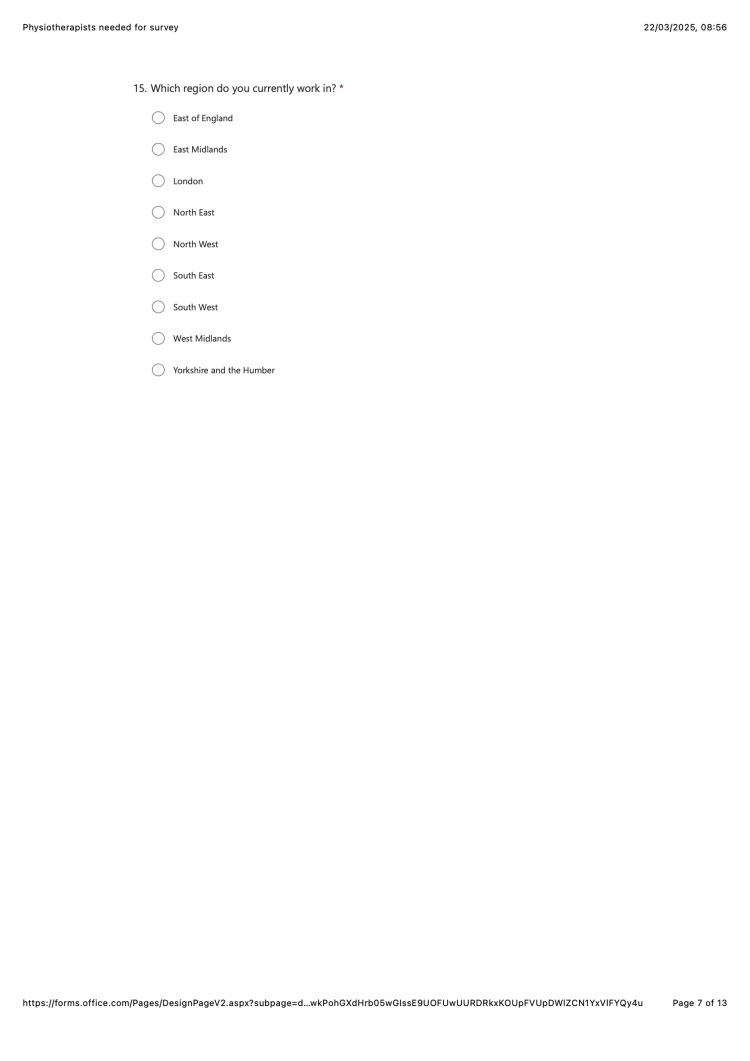
Survey page 7

**Figure 11:**
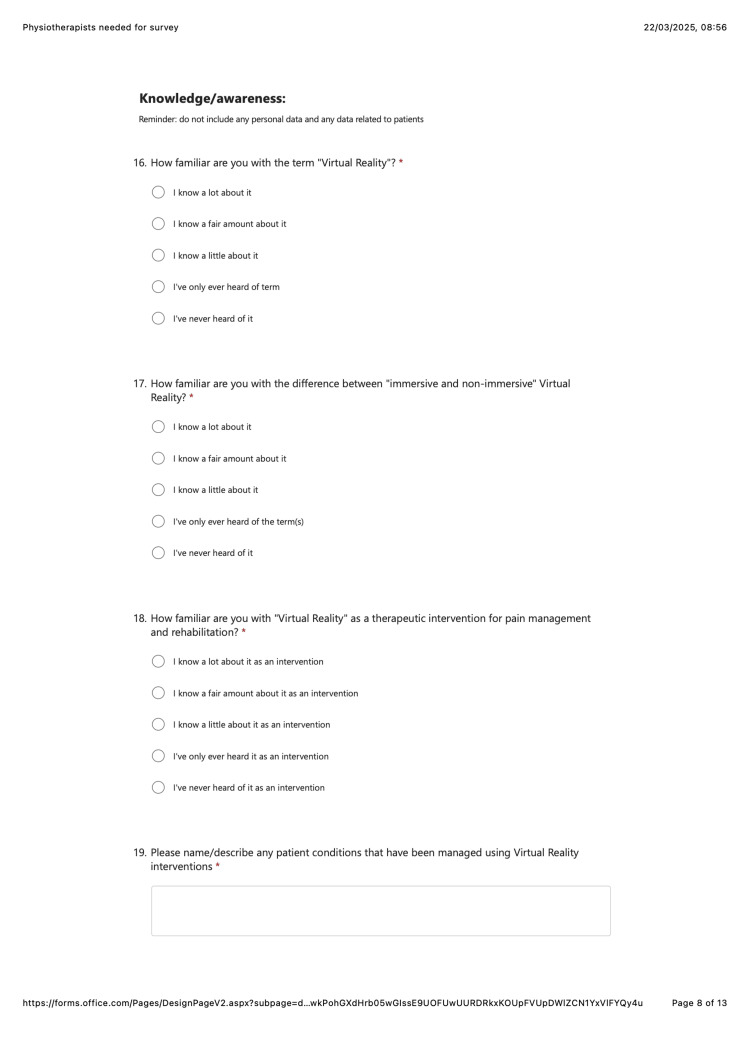
Survey page 8

**Figure 12:**
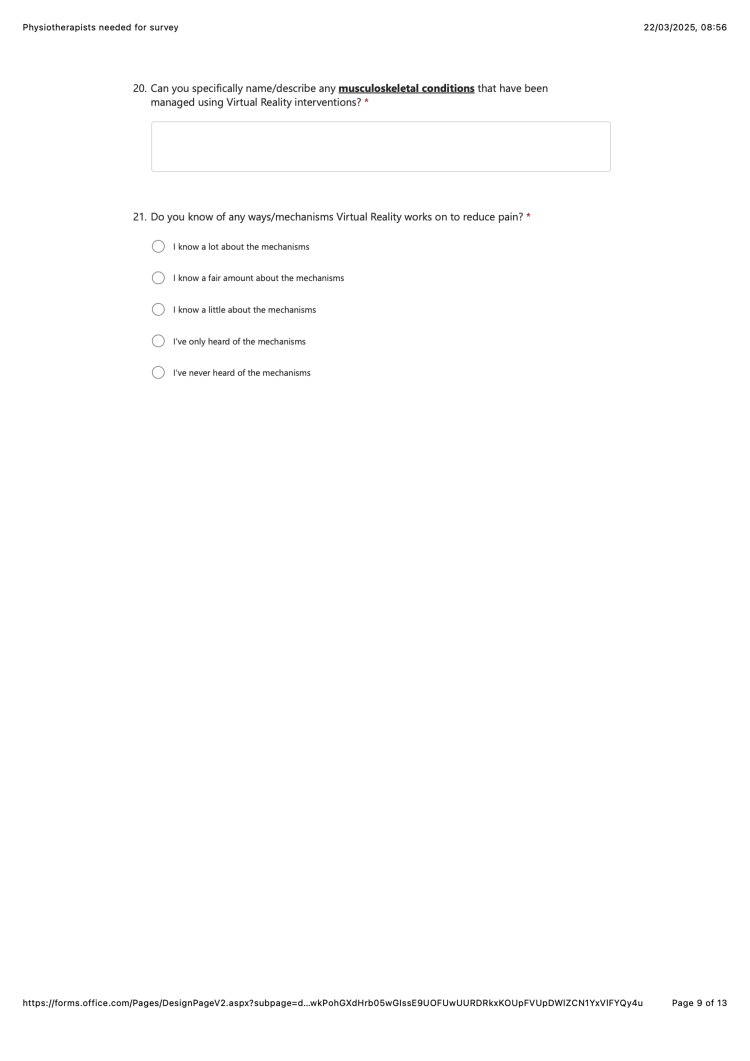
Survey page 9

**Figure 13:**
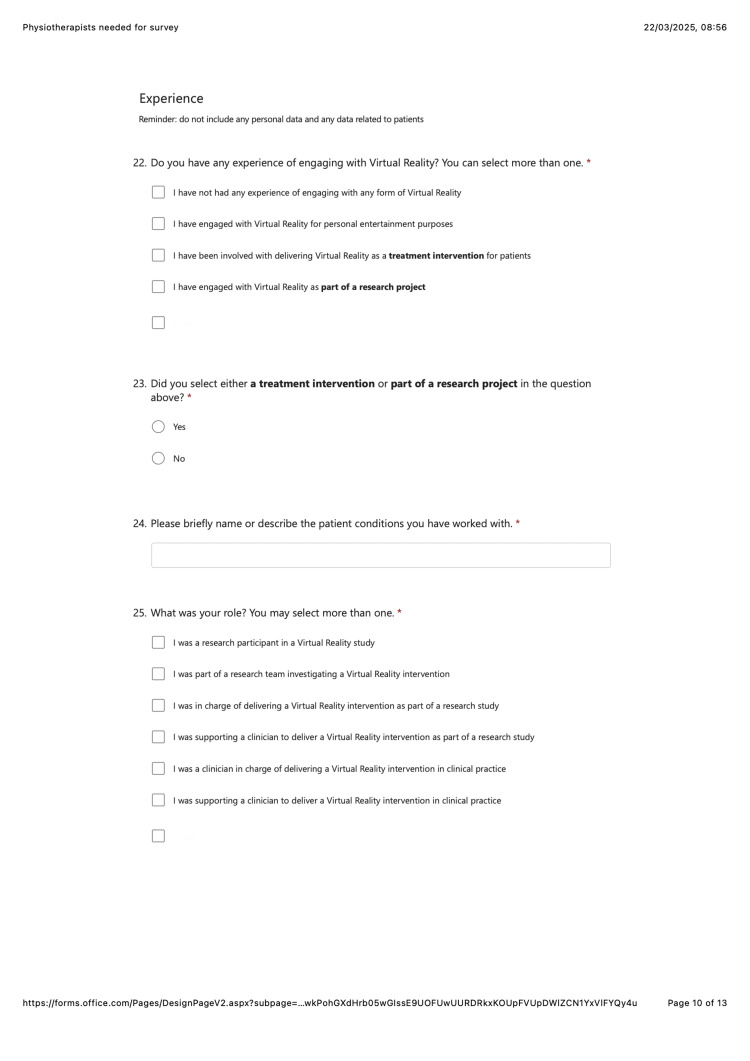
Survey page 10

**Figure 14:**
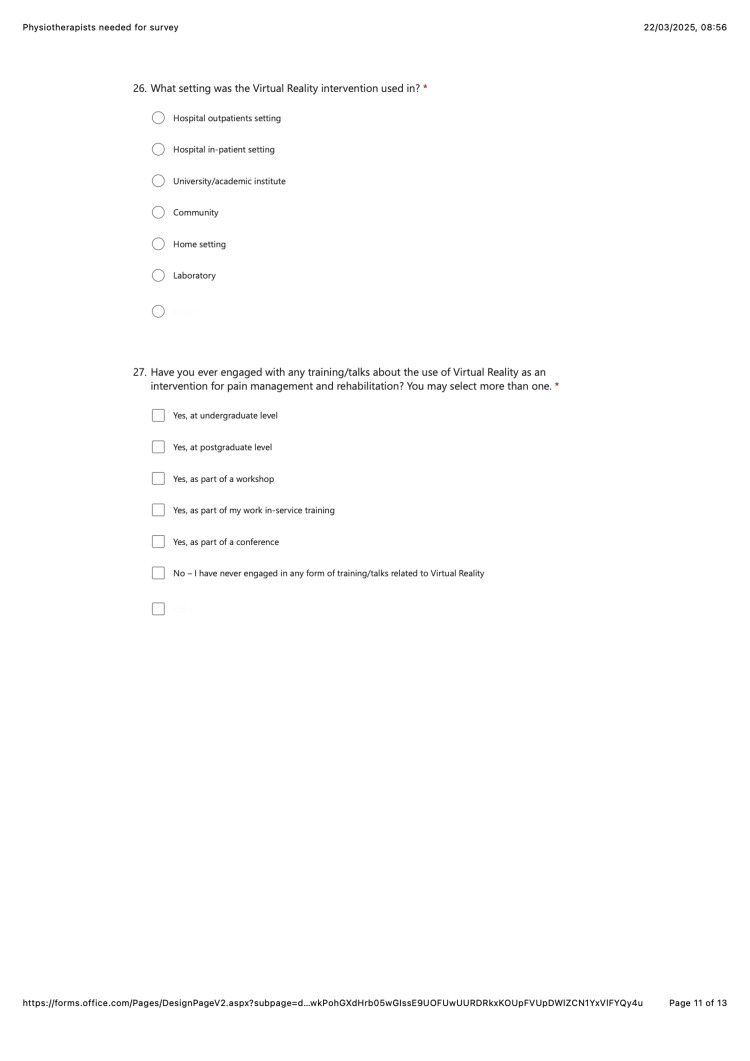
Survey page 11

**Figure 15:**
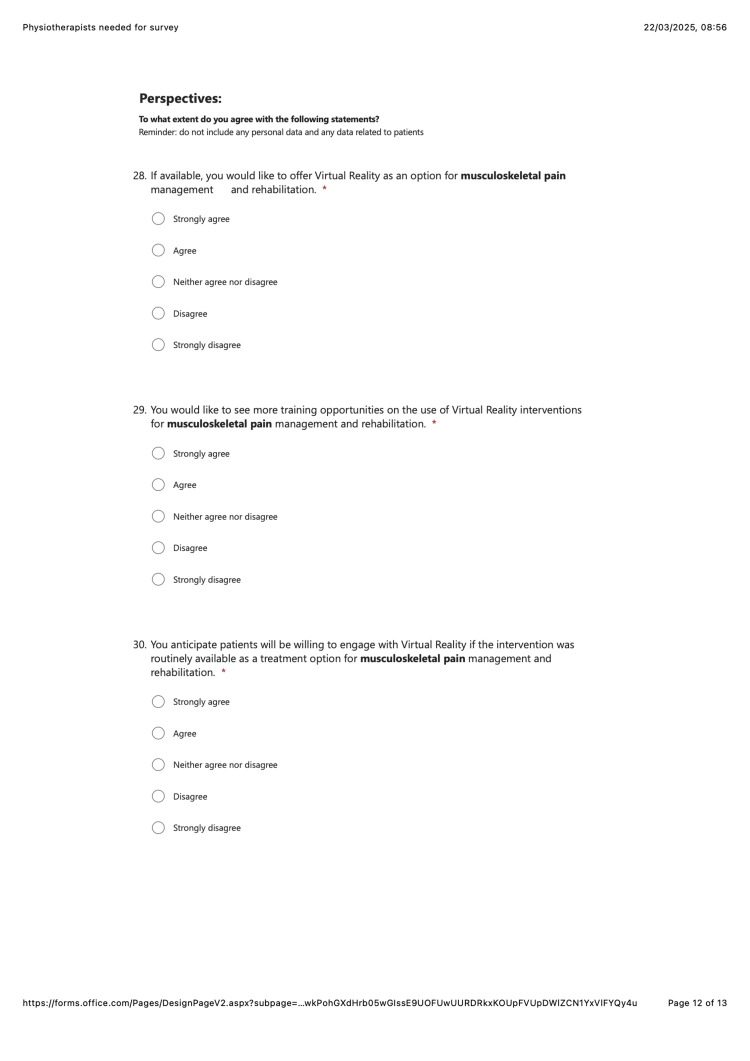
Survey page 12

**Figure 16:**
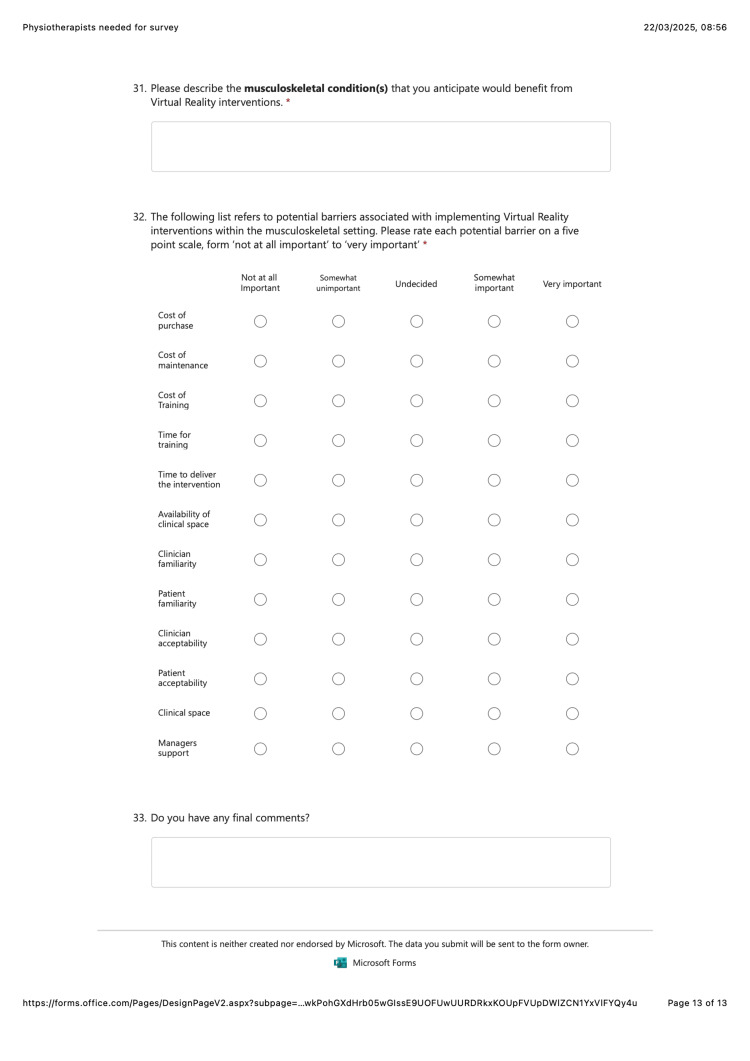
Survey page 13

**Figure 17:**
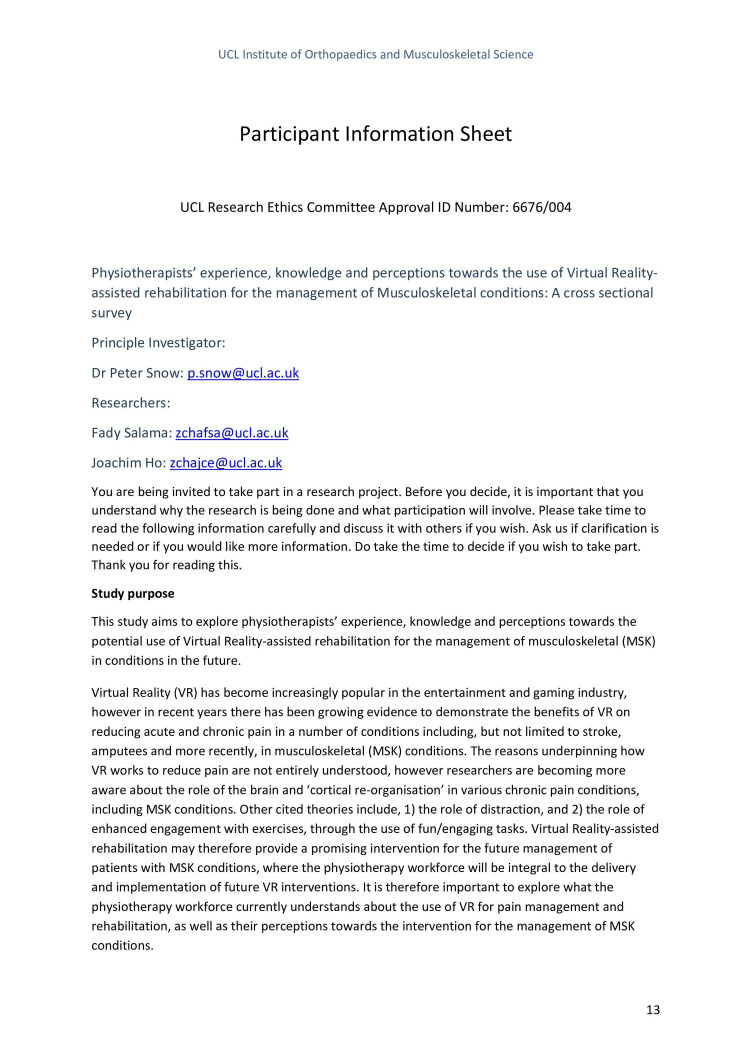
Participant Information Sheet

## Appendices

Appendix A

Appendix B
